# Fn14 Controls the SIRT2‐Mediated Deacetylation of Slug to Inhibit the Metastasis of Epithelial Ovarian Cancer

**DOI:** 10.1002/advs.202501552

**Published:** 2025-05-08

**Authors:** Anyue Wu, Shengze Li, Chunyang Feng, Ruiju He, Ruolan Wu, Zhijun Hu, Jinhua Huang, Wenjing Wang, Lei Huang, Lihua Qiu

**Affiliations:** ^1^ Department of Obstetrics and Gynecology Ren Ji Hospital School of Medicine Shanghai JiaoTong University Shanghai 200127 China; ^2^ Shanghai Key Laboratory of Gynecologic Oncology Shanghai 200127 China; ^3^ Department of Histoembryology Genetics and Developmental Biology Key Laboratory of Cell Differentiation and Apoptosis of Chinese Ministry of Education Shanghai Key Laboratory of Reproductive Medicine Shanghai Jiao Tong University School of Medicine Shanghai China; ^4^ State Key Laboratory of Systems Medicine for Cancer Shanghai Cancer Institute Ren Ji Hospital School of Medicine Shanghai Jiao Tong University Shanghai China

**Keywords:** deacetylation of slug, epithelial‐mesenchymal transition, Fn14, metastasis of epithelial ovarian cancer, post‐translational modifications

## Abstract

Metastatic spread of cancer is the leading cause of death in patients with epithelial ovarian cancer (EOC), and elucidation of the molecular mechanisms underlying this process is a major focus of cancer research. Fibroblast growth factor‐inducible 14 (Fn14) has been shown to regulate wound repair, inflammation, angiogenesis, and chemoresistance, but its functional role in metastasis in EOC is still unknown. Here it is reported that Fn14 is identified as a cancer metastasis suppressor that inhibits the migratory and invasive potential of EOC cells by down‐regulating epithelial‐mesenchymal transition (EMT). Mechanistically, it is identified that Fn14 promotes acetylation‐dependent protein degradation of Slug, a key transcriptional factor associated with EMT. The deacetylase Sirtuin 2 (SIRT2) has been reported to be involved in the deacetylation of Slug protein to stabilize it and then prevent its degradation in the nucleus. The results showed that Fn14 alters the subcellular localization of (SIRT2) by interacting with SIRT2, leading to reduced SIRT2 shuttling into the nucleus and subsequently promoting the acetylated degradation of Slug. Collectively, the work has demonstrated for the first time that Fn14 inhibits EOC metastasis by regulating SIRT2‐mediated Slug deacetylation, providing a new perspective and method for the development of future novel therapeutic strategies for the treatment of EOC metastasis.

## Graphical abstract

It is reported that Fn14 inhibited EOC metastasis by regulating Slug‐mediated EMT. Furthermore, Fn14 altered the subcellular localization of SIRT2 by interacting with SIRT2, leading to reduced SIRT2 shuttling into the nucleus and subsequently promoting the acetylated degradation of Slug.

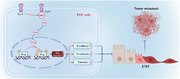

## 1 Introduction

Epithelial ovarian cancer (EOC) is the most deadly gynecological malignancy in women worldwide.^[^ ^1^ ^]^ Patients with EOC often present at a late stage with peritoneal dissemination and extensive metastases, with 5‐year survival rates ranging from 30–40%.^[^ ^2^ ^]^ Despite the therapeutic management of EOC, including surgical debulking and platinum‐based chemotherapy, that can control primary tumor growth, metastatic disease remains the greatest clinical challenge in oncology,^[^ ^3^, ^4^ ^]^ as these methods are still not very effective in preventing EOC metastasis. Therefore, a better understanding of the molecular mechanisms of EOC metastasis has the potential to significantly impact patient outcomes.

Fibroblast growth factor‐inducible 14 (Fn14), a type I transmembrane protein, is the cell surface receptor of tumor necrosis factor‐like weak inducer of apoptosis (TWEAK).^[^ ^5^, ^6^ ^]^ Fn14 is expressed in non‐lymphoid cells, including epithelial, endothelial, and mesenchymal cells.^[^ ^7^ ^]^ Fn14 is highly conserved from an evolutionary standpoint and has been shown to play multiple roles in the process of wound repair,^[^ ^8^ ^]^ and inflammation by regulating pro‐inflammatory cytokine secretion.^[^ ^9^ ^]^ Nowadays, the biological function of Fn14 in tumor metastasis is being unraveled, which is crucial for controlling cytoskeleton modulation, extracellular matrix degradation, apoptosis, and angiogenesis.^[^ ^10^, ^11^ ^]^ And evidence has shown that the tumor necrosis factor receptor‐associated factor and nuclear factor kappa B signaling pathways are considered two main downstream pathways activated by Fn14 signaling.^[^ ^12^ ^]^ Our previous study identified that overexpression of Fn14 enhances cisplatin‐induced apoptosis and alleviates cisplatin resistance through modulation of the ubiquitylation and degradation of TP53‐R248Q protein in high‐grade serous ovarian cancer,^[^ ^13^ ^]^ however, the role of Fn14 in the metastasis of EOC has not been described.

In this study, we explored the functional role of Fn14 and its underlying mechanism in EOC metastasis in vivo and in vitro. We demonstrate for the first time that Fn14 attenuates metastasis by inhibiting epithelial‐mesenchymal transition (EMT) in EOC. EMT is a process in which epithelial cells acquire mesenchymal features. In EOC, EMT is associated with tumor initiation, invasion, metastasis, and resistance to therapy.^[^ ^14^ ^]^ We then investigated the mechanism by which Fn14 inhibits EMT in EOC and found that Fn14 promotes the degradation of the Slug transcription factor associated with EMT. Post‐translational modification is important in the regulation of Slug protein stability and degradation.^[^ ^15^, ^16^ ^]^ Sirtuin 2 (SIRT2), a nicotinamide adenine dinucleotide (NAD+)‐dependent deacetylase,^[^ ^17^ ^]^ was found to deacetylate the Slug protein to stabilize it in the nucleus.^[^ ^18^ ^]^ Furthermore, we found that by interacting with SIRT2, Fn14 can prevent SIRT2 shuttling into the nucleus, leading to a change in the subcellular localization of SIRT2 and subsequently promoting the acetylated degradation of Slug. Our results highlight the importance of Fn14 in the regulation of Slug degradation mediated by SIRT2 in EOC, suggesting that Fn14 may act as a therapeutic target to suppress metastasis and improve the prognosis of EOC patients.

## 2 Results

### 2.1 Loss of Fn14 Correlates with Metastasis and Poor Prognosis of EOC

**Figure 1:**
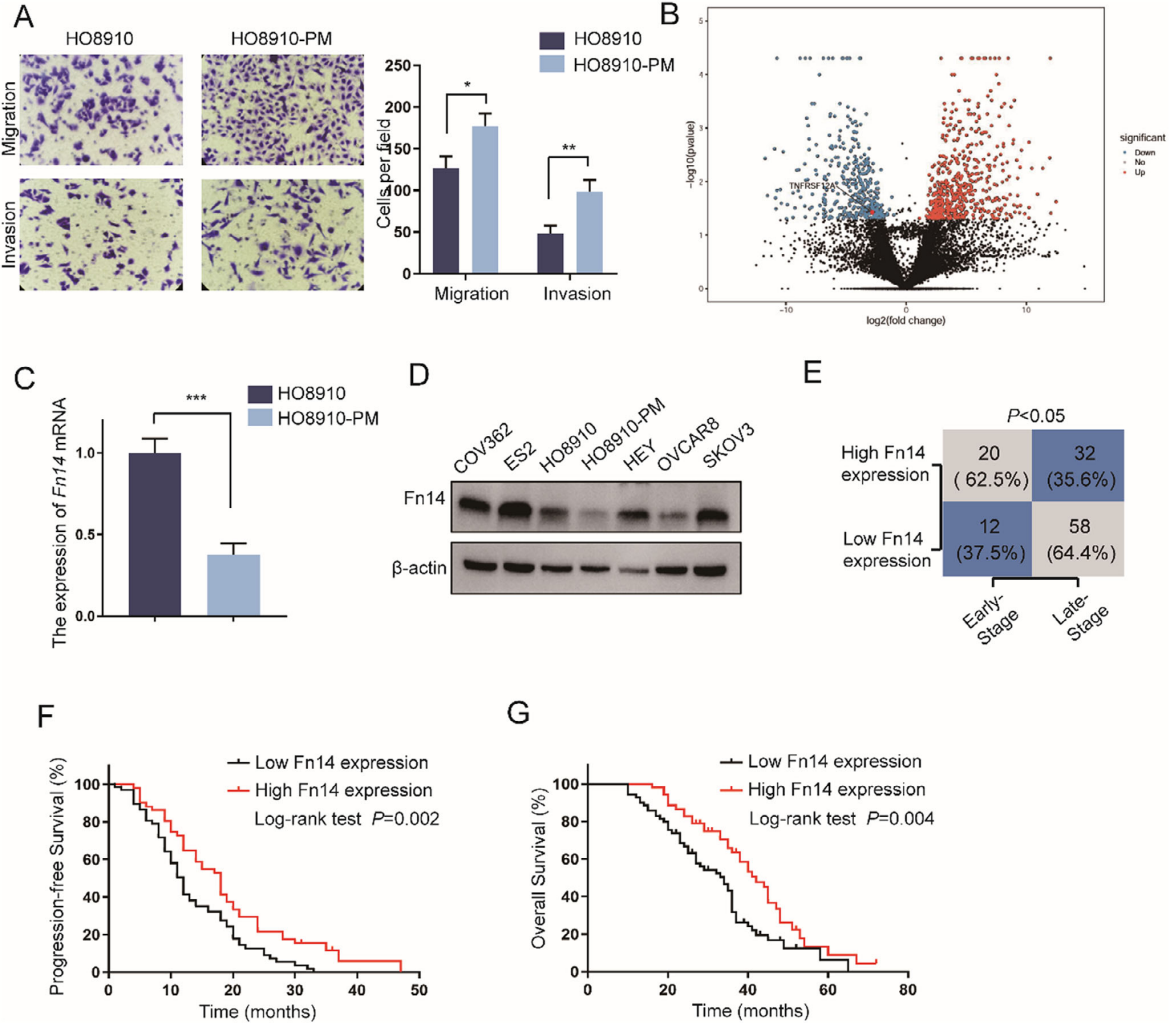
Loss of Fn14 correlates with metastasis and poor prognosis of EOC. A) The ability of invasion and migration were examined by transwell assays in HO8910 and HO8910‐PM cells (n = 3 per cell line, scale bar  =  100 µm). B) RNA‐seq analysis of HO8910 versus HO8910‐PM cells and volcano plot showing differentially expressed genes. C) RT‐qPCR detecting the expression of *Fn14* mRNA in HO8910 and HO8910‐PM cells (n = 3). D) Western blot analysis detecting the expression of Fn14 in EOC cells. E)Distribution of Fn14 expression in early‐stage group and late‐stage group. F,G) Survival outcome analysis showed that EOC patients with overexpression of Fn14 exhibited better progression‐free survival and overall survival compared with those who had low expression of Fn14. Unpaired Student's *t*‐test was performed for comparison between two groups, one‐way ANOVA was performed for multiple group comparisons, and the Chi‐square test was applied for comparisons of proportions. ^*^ *p* < 0.05, ^**^ *p* < 0.01, ^***^ *p* < 0.001.

HO8910 and HO8910‐PM cells are EOC cell lines with different metastatic potentials, with HO8910 representing a low metastatic potential cell line and HO8910‐PM serving as its paired high metastatic potential counterpart. As shown in **Figure** **1**A, HO8910‐PM showed a high metastatic capacity compared with the parental cell (HO8910). And then, we performed RNA sequencing (RNA‐seq) analysis on these cell lines to identify potential regulatory factors governing EOC metastasis. And the result of RNA‐seq showed that a total of 652 genes were differentially expressed according to fold‐change (FC) filtering (|log2FC|>1) and *p*‐value <0.05. Among them, we found that Fn14 is significantly downregulated in HO8910‐PM compared with HO8910 (Figure 1B). In addition, our previous study demonstrated that Fn14 overcomes cisplatin resistance of high‐grade serous ovarian cancer by promoting Mdm2‐mediated p53‐R248Q ubiquitination and degradation,^[^ ^13^ ^]^ therefore, we supposed that Fn14 might play a crucial role in regulating metastasis of EOC. To explore the function of Fn14 in the metastasis of EOC, we measured the expression of Fn14 in HO8910 cells and HO8910‐PM cells. The results showed that reduced expression of *Fn14* mRNA was observed in HO8910‐PM cells compared to HO8910 cells (Figure 1C). Next, we examined Fn14 expression in EOC cell lines and found the expression of Fn14 protein is decreased in HO8910‐PM cells compared to HO8910 cells and ES2 cells exhibit the highest endogenous expression of Fn14 (Figure 1D). Considering the aforementioned results, we chose HO8910, HO8910‐PM, and ES2 cells for subsequent experiments. We further evaluated the expression of Fn14 in a panel of 122 EOC cases by IHC (Figure S1A, Supporting Information) and analyzed the correlation between Fn14 expression and clinicopathological characteristics of EOC. As shown in Figure S1 and Table S1 (Supporting Information), the Fn14 protein expression level did not correlate to patient age, histology, and pathological type. Fn14 expression in early‐stage (I‐II) patients was significantly higher than in patients with late‐stage (III‐IV) (*p*<0.05) (Figure 1E). Moreover, lymph node metastasis was seen more in the low Fn14 expression group compared to the high Fn14 expression group (*p*<0.01). Survival analysis revealed that low Fn14 expression is significantly associated with poor progression‐free survival (*p*<0.01) and overall survival (*p*<0.01) (Figure 1F,G).

In addition, analysis by the multivariate Cox proportional hazards model determined that the high expression of Fn14 was not associated with an increased progression‐free survival (PFS). But, for those patients with high Fn14 expression, the overall survival (OS) was significantly higher (Figure S1 and Tables S2 and S3, Supporting Information) (HR 0.622, *p*<0.05). Furthermore, it was clearly observed that other factors were independently related with longer PFS and OS, such as early‐stage disease and low‐grade EOC (Figure S1 and Tables S2 and S3, Supporting Information). These collective data indicated that down‐regulated Fn14 might be correlated with poor clinical features, including metastasis, in EOC patients.

### 2.2 Fn14 Alleviates the Metastasis of EOC Cells In Vitro

**Figure 2:**
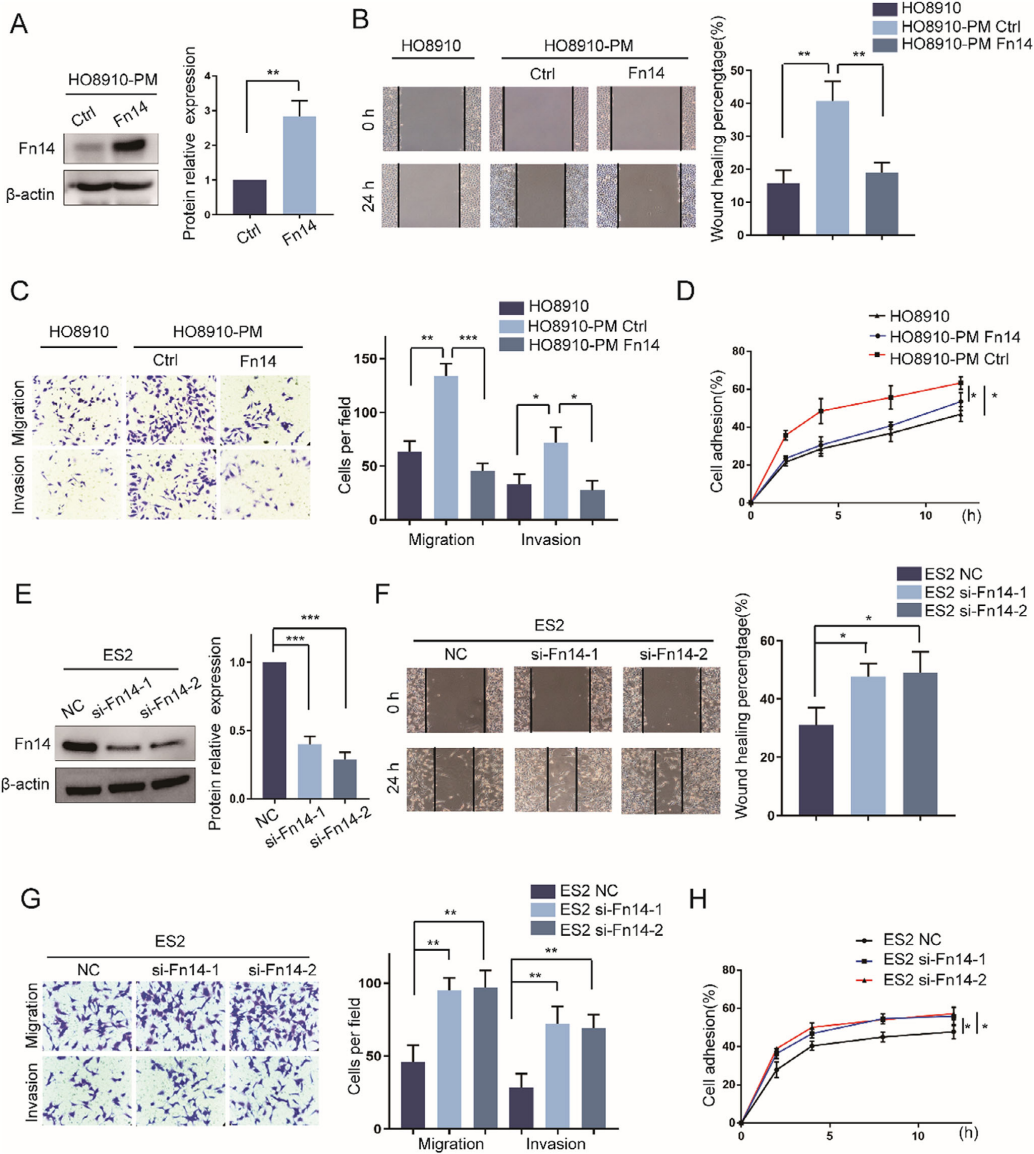
Fn14 alleviates the metastasis of EOC cells in vitro. A) Western blot analysis detecting Fn14 expression in HO8910‐PM cells infected with Fn14 lentivirus (n = 3). B,C) Cell migration and invasion were examined by wound healing assay and transwell assay in HO8910 cells and HO8910‐PM cells infected with Fn14 lentivirus (n = 3 per cell line, Transwell scale bar  =  100 µm, Wound healing scale bar  =  200 µm). D) CCK‐8 assay was used to detect cell adhesion in HO8910 cells and HO8910‐PM cells infected with Fn14 lentivirus (n = 3). E) Western blot analysis detecting Fn14 expression in ES2 cells transfected with the Fn14 siRNA (n = 3). F,G) Cell migration and invasion were examined by wound healing assay and transwell assay in ES2 cells transfected with the Fn14 siRNA (n = 3 per cell line, Transwell scale bar  =  100 µm, Wound healing scale bar  =  200 µm). H) CCK‐8 assay was used to detect cell adhesion in ES2 cells transfected with the Fn14 siRNA(n = 3). Unpaired Student's *t*‐test was performed for comparison between two groups, one‐way ANOVA was performed for multiple group comparisons, and the Chi‐square test was applied for comparisons of proportions. ^*^ *p* < 0.05, ^**^ *p* < 0.01, ^***^ *p* < 0.001.

To investigate whether Fn14 could be sufficient to inhibit the capacity of metastasis in EOC cells, Fn14 was stably overexpressed in HO8910‐PM (**Figure** **2**A). The wound healing assay showed a significant reduction in the migration of HO8910‐PM cells with Fn14 overexpression (Figure 2B). And transwell assays showed increased Fn14 expression markedly reduced HO8910‐PM cell migration and invasion (Figure 2C). Furthermore, the adhesion assay showed that increased Fn14 expression remarkably attenuated the ability of adhesiveness of HO8910‐PM cells (Figure 2D). To further confirm the role of Fn14 in the regulation of metastasis, the expression of Fn14 was knocked down in ES2 cells (Figure 2E). We observed that knockdown of Fn14 promotes a significant increase in the migration of ES2 cells (Figure 2F) and transwell assays also showed knockdown of Fn14 promotes an increase in the migration and invasion of ES2 cells (Figure 2G). Additionally, the knockdown of Fn14 promotes the ability of adhesiveness in ES2 cells (Figure 2H). Taken together, these data indicated that Fn14 could inhibit the malignant phenotype of EOC cells by suppressing metastasis.

### 2.3 Fn14 Attenuates Metastasis of EOC Cells by Regulating EMT

**Figure 3:**
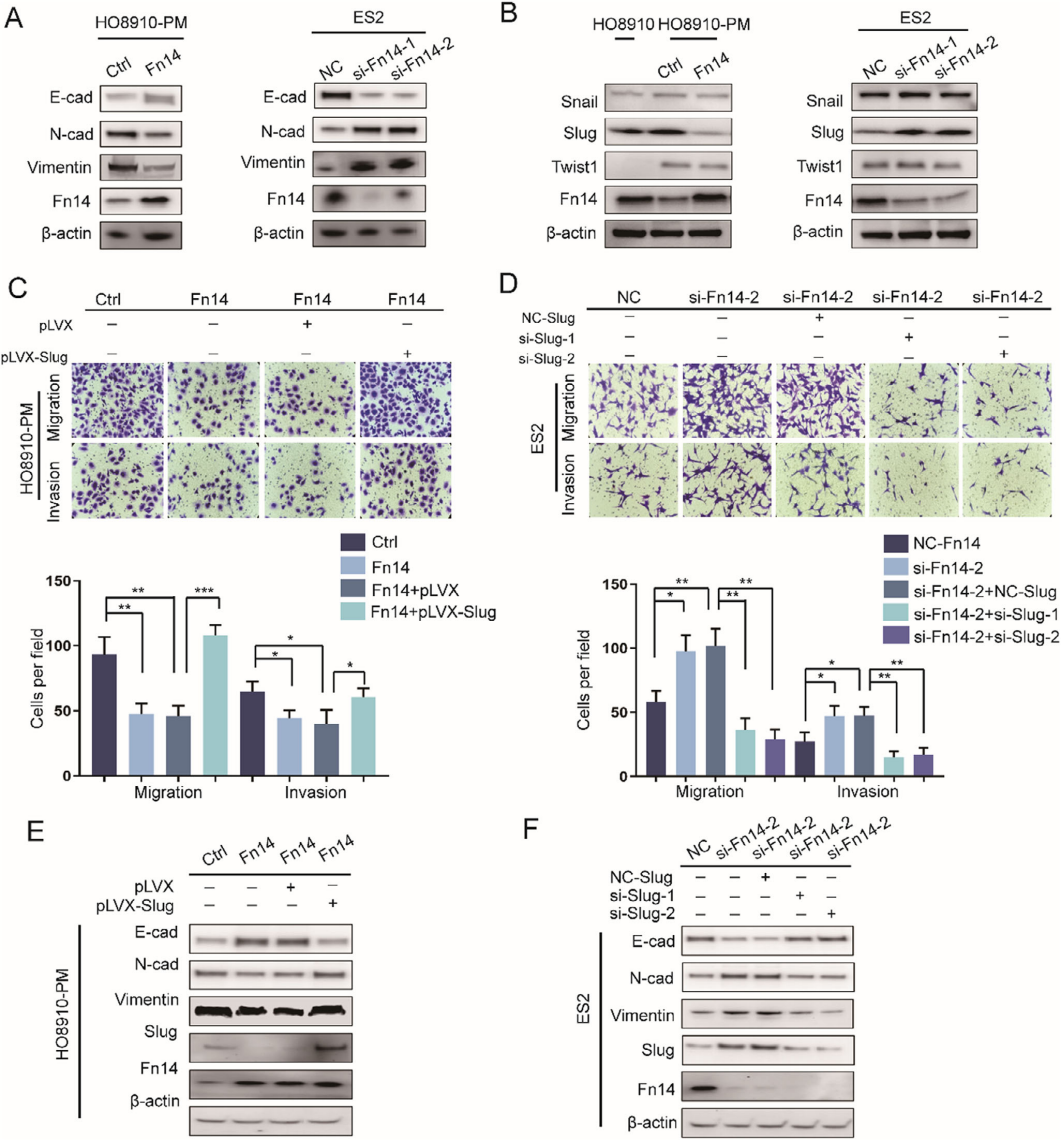
Fn14 attenuates metastasis of EOC cells by regulating EMT. A) Western blot analysis detecting the EMT makers in HO8910‐PM and ES2 cells with specific treatment (n = 3). B) Western blot analysis detecting the transcription activator of EMT in HO8910‐PM and ES2 cells with specific treatment (n = 3). C) The ability of migration and invasion was restored by transduction with Slug ectopically expressing plasmid in Fn14‐overexpression HO8910‐PM cells (n = 3 per cell line, scale bar  =  100 µm). D) The ability of migration and invasion was decreased by transduction with Slug siRNA in Fn14‐knockout ES2 cells (n = 3 per cell line, scale bar  =  100 µm). E) The expression of EMT markers was restored by transduction with Slug ectopically expressing plasmid in Fn14‐overexpression HO8910‐PM cells (n = 3). F) The expression of EMT markers was down‐regulated by transduction with Slug siRNA in Fn14‐knockout ES2 cells(n = 3). Unpaired Student's *t*‐test was used for comparison between two groups, one‐way ANOVA for multiple group comparisons. ^*^ *p* < 0.05, ^**^ *p* < 0.01, ^***^ *p* < 0.001.

During the cell culture process, we found the morphology of HO8910‐PM was changed and partial epithelial cells transdifferentiated into motile mesenchymal cells (Figure S2A, Supporting Information). EMT is a key mechanism involved in cancer invasion and metastasis,^[^ ^19^ ^]^ therefore, we hypothesized that Fn14 might inhibit metastasis of EOC cells by regulating EMT. And then, we found overexpression of Fn14 could reverse EMT in HO8910‐PM (Figure S2A, Supporting Information). Next, we detected the expression of EMT‐related markers by western blot in EOC cells. As a result, overexpression of Fn14 was associated with the induction of the epithelial marker E‐cadherin (E‐cad) and loss of expression of mesenchymal markers N‐cadherin (N‐cad) and Vimentin (**Figure** **3**A; Figure S2B, Supporting Information). Next, Fn14 expression was down‐regulated in ES2 cells, accompanied with the suppression of E‐cad and the overexpression of N‐cad, Vimentin (Figure 3A; Figure S2B, Supporting Information). EMT is mediated by key transcription factors, including the Snail family, TWIST1, and basic helix‐loop‐helix transcription factors and we found overexpression Fn14 could down‐regulate the expression of Slug (Snail2) in HO8910‐PM cells and knockdown of Fn14 could up‐regulate the expression of Slug in ES2 cells (Figure 3B; Figure S2C, Supporting Information). To further determine whether Slug contributed to the Fn14‐mediated suppression of EOC cells, therefore, Slug was up‐regulated in HO8910‐PM cells with Fn14 overexpression leading to the abrogation of Fn14‐mediated metastasis (Figure 3C) and Slug was down‐regulated in ES2 cells with Fn14 knockdown resulted in the abrogation of Fn14‐mediated metastasis (Figure 3D). Moreover, the re‐expression of Slug in the Fn14 overexpression HO8910‐PM cells restored the mesenchymal makers’ expression (Figure 3E; Figure S2D, Supporting Information), and the deletion of Slug expression in the Fn14 knockdown ES2 cells re‐established the epithelial makers’ expression (Figure 3F; Figure S2E, Supporting Information). These data suggested that Fn14 inhibits the invasion and metastasis of EOC cells by down‐regulation of Slug expression.

### 2.4 Fn14 Promotes the Acetylation‐Dependent Degradation of Slug Protein

**Figure 4:**
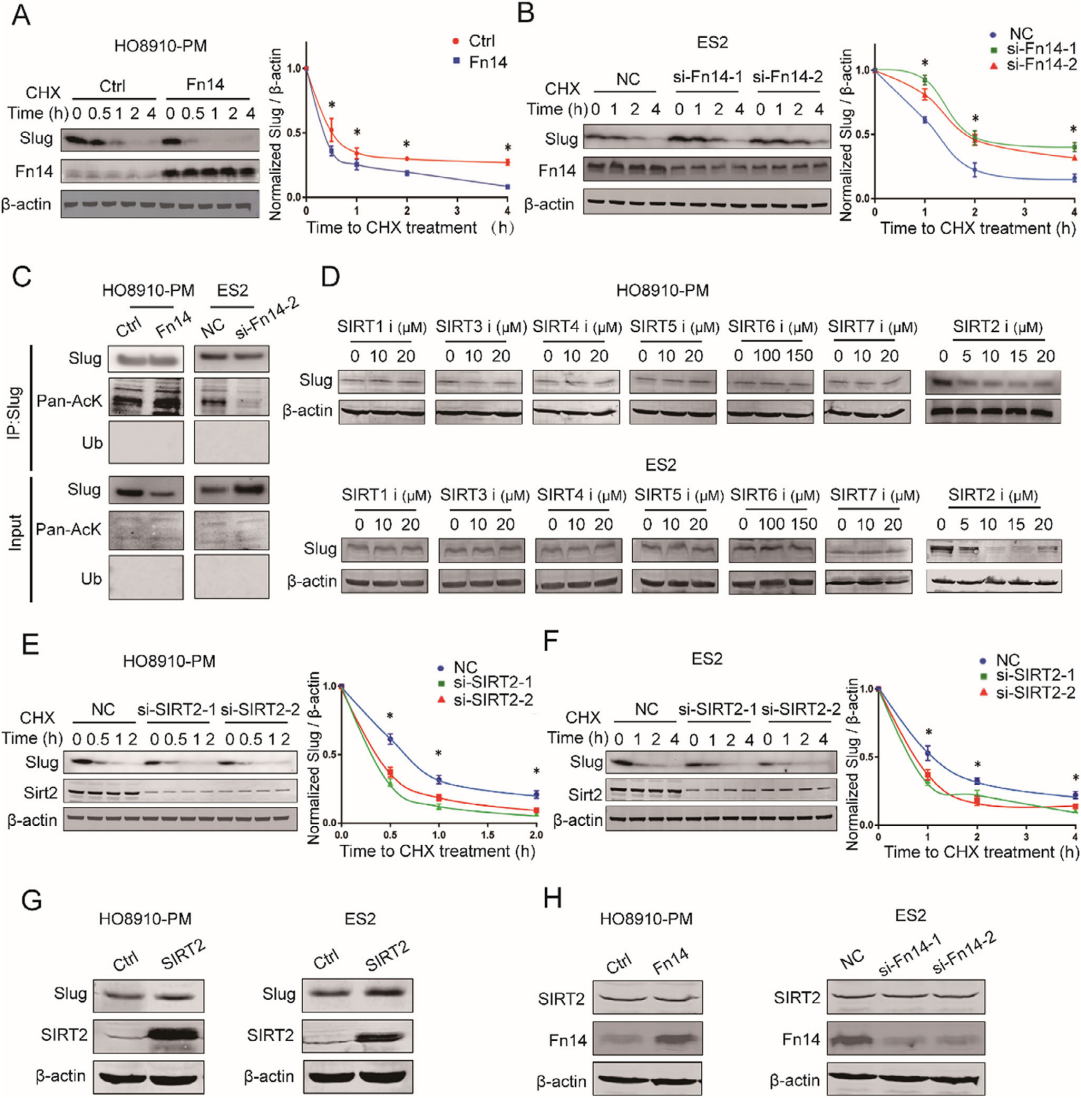
Fn14 promotes the acetylation‐dependent degradation of Slug protein. A) Upon CHX treatment, western blot analysis detected the expression of Slug in HO8910‐PM cells after infection with Fn14 lentivirus (n = 3). B) Upon CHX treatment, western blot analysis detected the expression of Slug in ES2 cells after transfected with the Fn14 siRNA (n = 3). C) Co‐IP analysis detecting the level of ubiquitination and acetylation of Slug in HO8910‐PM and ES2 cells with specific treatment (n = 3). D) Western blot analysis was performed to detect the expression of Slug in EOC cells with selective inhibitors of Sirt family proteins (Selisistat, SIRT1inhibitor; AGK2, SIRT2 inhibitor; 3‐TYP, SIRT3 inhibitor; SIRT4‐IN‐1, SIRT4 inhibitor; SIRT5 inhibitor 4, SIRT5 inhibitor; OSS_128167, SIRT6 inhibitor; SIRT7 inhibitor 97491, SIRT7 inhibitor). E,F) Western blot analysis was performed to examine the expression of Slug in EOC cells transfected with the SIRT2 siRNA (n = 3). G) Western blot analysis was performed to examine the expression of Slug in EOC cells transfected with SIRT2 ectopically expressing plasmid(n = 3). H)Western blot analysis detecting the level of SIRT2 in HO8910‐PM and ES2 cells with up‐ or down‐regulated expression of Fn14 (n = 3). Unpaired Student's *t*‐test was used to compare two groups and one‐way ANOVA was used to compare multiple groups. ^*^ *p* < 0.05, ^**^ *p* < 0.01, ^***^ *p* < 0.001.

Next, we were trying to explain the mechanism of Fn14 down‐regulation of the Slug expression in EOC cells. We have previously demonstrated that Fn14 downregulates Slug protein expression, and we further investigated its regulatory effect on Slug transcriptional output by quantifying mRNA levels. The results showed that overexpression of Fn14 up‐regulated the *Slug* mRNA (Figure S3A, Supporting Information). And knockdown of Fn14 by siRNA‐1 up‐regulated *Slug* mRNA, but the knockdown of Fn14 by siRNA‐2 down‐regulated *Slug* mRNA (Figure S3A, Supporting Information). This discordant regulatory effect of Fn14 on *Slug* mRNA levels versus protein expression suggests that Fn14 might downregulate Slug expression at the post‐translational level. Upon cycloheximide (CHX) blockade of de novo protein synthesis, overexpression of Fn14 could promote the degradation of Slug and lead to shortening protein half‐life of Slug in HO8910‐PM cells (**Figure** **4**A). Conversely, Fn14 knockdown inhibited the degradation of Slug and resulted in extending the protein half‐life of Slug in ES2 cells (Figure 4B). Given the fact that the proteolytic turnover of Slug is regulated by post‐translational modifications such as ubiquitination and acetylation,^[^ ^20^ ^]^ we therefore determined the ubiquitination and acetylation levels of the Slug protein in EOC cells. Immunoprecipitation assays failed to detect ubiquitinated Slug protein in EOC cells, and Fn14 had no detectable effect on Slug ubiquitination levels, but the amount of endogenous acetylated Slug was significantly increased in HO8910‐PM cells overexpressing Fn14, and Fn14 knockdown inhibited Slug acetylation in ES2 cells (Figure 4C). Extensive studies have clearly revealed that SIRTs constitute a class of proteins with deacetylase activity and seven SIRT family members have been identified in mammals, from SIRT1 to SIRT7.^[^ ^21^ ^]^ And we found that Slug protein abundance is regulated by SIRT2 in EOC, while other members of the SIRT family had no significant effect on Slug protein levels (Figure 4D), consistent with previous reports. To confirm degradation of Slug was regulated by SIRT2 in EOC, the expression of SIRT2 was down‐regulated in HO8910‐PM and ES2 cells and resulted in shorting the half‐life of Slug protein (Figure 4E,F). Similar results were obtained in the EOC cells treated with AGK2 (inhibitors of SIRT2) (Figure S3B,C, Supporting Information). Furthermore, overexpression SIRT2 could promote the expression of Slug in EOC cells (Figure 4G; Figure S3D, Supporting Information). In addition, we investigated the effect of mutating the K116 site of Slug on protein stability in EOC and showed that the K116Q mutation significantly promotes Slug protein degradation compared with Slug WT (Figure S3F, Supporting Information). To determine whether SIRT2 expression is affected by Fn14 protein levels, we examined the expression of SIRT2 following Fn14 perturbation. In Fn14 overexpression cells where Slug protein levels remained unchanged and knockdown of Fn14 expression had no effect on SIRT2 protein levels in ES2 cells (Figure 4H; Figure S3E, Supporting Information). Collectively, we established the hypothesis that Fn14 could mediate Slug acetylation and the underlying mechanism might be related with regulating SIRT2.

### 2.5 Fn14 Enhances Acetylation and Degradation of Slug by Interaction with SIRT2

**Figure 5:**
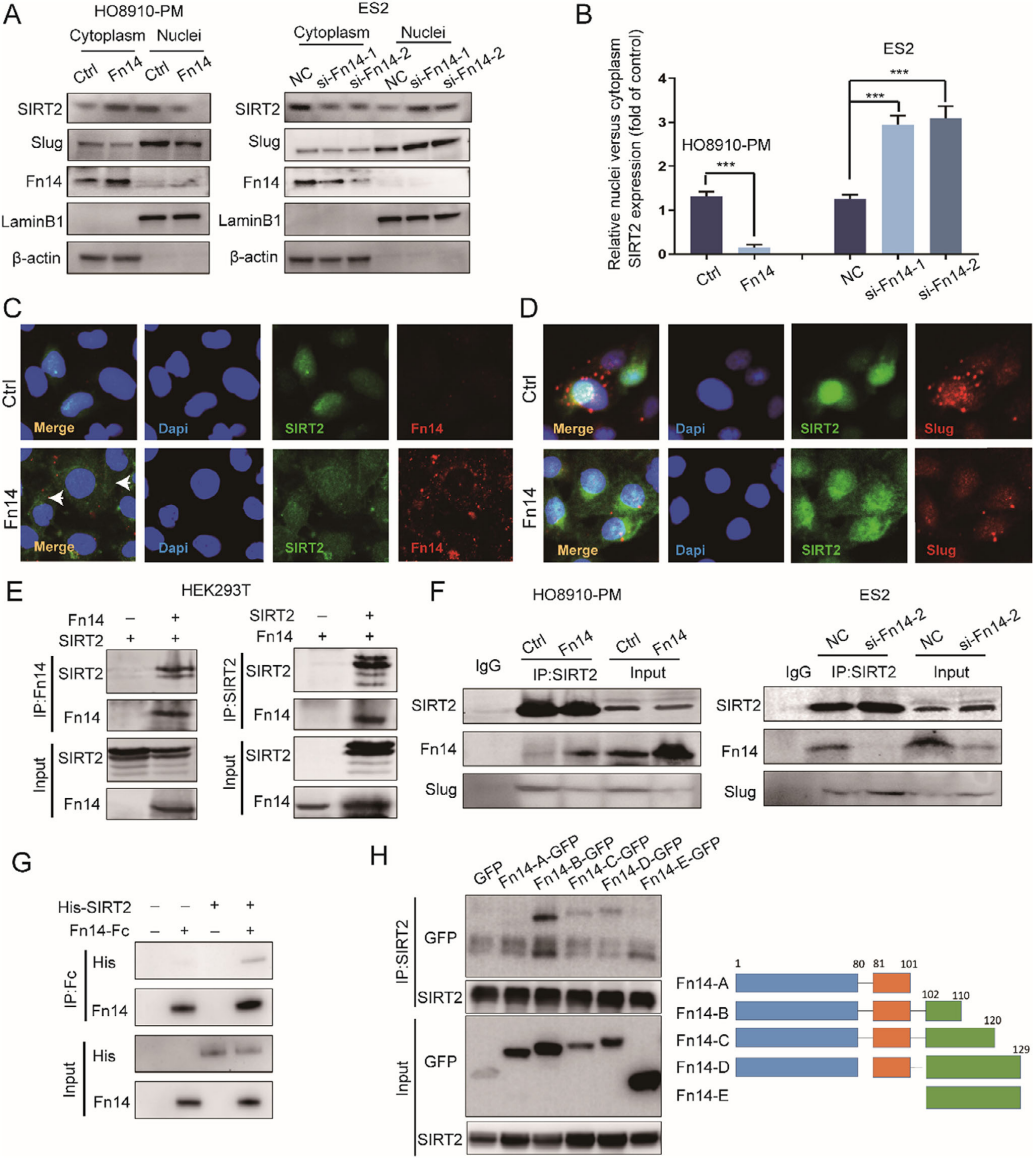
Fn14 enhances acetylation and degradation of Slug by interaction with SIRT2. A,B) Detecting the distribution of SIRT2 and Slug in cytoplasm or nucleus in EOC cells with up‐ or down‐regulated expression of Fn14 by western blot analysis (n = 3). C) Immunofluorescence of Fn14 (red) and SIRT2 (green) in HO8910‐PM cells infected with Fn14 lentivirus. DAPI (blue) was used to visualize the nucleus (Scale bar = 20 µm). D) Immunofluorescence of Slug (red) and SIRT2 (green) in HO8910‐PM cells infected with Fn14 lentivirus (Scale bar = 20 µm). E) Lysates from HEK293T cells transfected with Fn14 and SIRT2 were subjected to anti‐Fn14 or anti‐SIRT2 immunoprecipitation. Immunoblots of Fn14 and SIRT2 are shown (n = 3). F) Lysates from HO8910‐PM and ES2 with w‐specific treatment were subjected to anti‐SIRT2 immunoprecipitation. Immunoblots of endogenous Fn14 and Slug are shown (n = 3). G) Interaction between recombinant Fn14 and SIRT2 was detected by pull‐down analysis (n = 3). H) Lysates from HEK293T cells transfected with GFP‐tagged truncation of Fn14 and SIRT2 were subjected to anti‐SIRT2 immunoprecipitation. Immunoblots of GFP are shown (n = 3). Unpaired Student's t‐test was used to compare two groups, one‐way ANOVA was used to compare multiple groups. ^*^ *p* < 0.05, ^**^ *p* < 0.01, ^***^ *p* < 0.001.

We previously found Fn14 markedly accelerates acetylation and degradation of Slug but Fn14 just has a slight impact on the expression of SIRT2. Thus, we supposed there might exist an unknown mechanism of SIRT2 regulated by Fn14. SIRT2 is a shuttle protein moving between the cytoplasm and the nucleus. And SIRT2 regulates the deacetylation of target protein on the condition that SIRT2 needs to shuttle into the nucleus.^[^ ^22^ ^]^ This evidence enlightened us that Fn14 might affect the subcellular localization of SIRT2. Overexpression of Fn14 increased the SIRT2 expression located in the cytoplasm and decreased SIRT2 shuttling into the nucleus in HO8910‐PM cells (**Figure** **5**A,B). Conversely, the knockdown of Fn14 reduced SIRT2 expression located in the cytoplasm and promoted SIRT2 shuttling into the nucleus in ES2 cells (Figure 5A,B). Nucleus‐cytoplasmic shuttling of SIRT2 was regulated by nuclear export in a Crm1‐dependent manner.^[^ ^23^ ^]^ Notably, inhibition of CRM1‐mediated nuclear export by leptomycin B blocked Fn14‐mediated cytoplasmic accumulation of SIRT2(Figure S4A,B, Supporting Information). And then, IF staining results further validated that SIRT2 is co‐localized with Fn14 on the cytomembrane and overexpression of Fn14 did decrease SIRT2 shuttling into the nucleus in HO8910‐PM cells (Figure 5C). Moreover, overexpression of Fn14 reduced the co‐localization between SIRT2 and Slug in the nucleus (Figure 5D). We next sought to determine whether Fn14 might interact with SIRT2. Reciprocal co‐IP was performed in HEK293T cells that ectopically expressed Fn14 and SIRT2 and the data showed Fn14 could interact with SIRT2 (Figure 5E). Likewise, endogenous interaction between Fn14 and SIRT2 was observed in HO8910‐PM and ES2 cells (Figure 5F). In addition, Fn14 overexpression reduced SIRT2 interaction with Slug, and Fn14 knockdown promoted SIRT2 interaction with Slug in EOC cells (Figure 5F). To further validate our findings, a pull‐down assay further confirmed that a direct physical interaction between Fn14 and SIRT2 (Figure 5G). To identify the interaction domain of Fn14 binding to SIRT2, we carried out co‐IP with different domains of Fn14. Fn14 structure can be divided into three parts, the N‐terminal extracellular region(1–80aa), the transmembrane region(81–101aa), and the C‐terminal intracellular region(102–129aa), we established five truncations of Fn14 with GFP‐tag: Fn14‐A‐GFP: 1–101aa, Fn14‐B‐GFP: 1–110aa, Fn14‐C‐GFP: 1–120aa, Fn14‐D‐GFP: 1–129aa and Fn14‐E‐GFP: 102–129aa. The five Fn14 truncations were co‐transfected into HEK 293T cells with SIRT2. The co‐IP result showed that SIRT2 binds to the domain aa 102 to 110 in the C‐terminal intracellular region of Fn14 (Figure 5H). Together, these results show that Fn14 interacts with SIRT2 protein leading to a decrease in SIRT2 shuttling into the nucleus and the loss of Slug stabilization.

### 2.6 Silencing SIRT2 Inhibits the Invasion and Metastasis of EOC Cells

**Figure 6:**
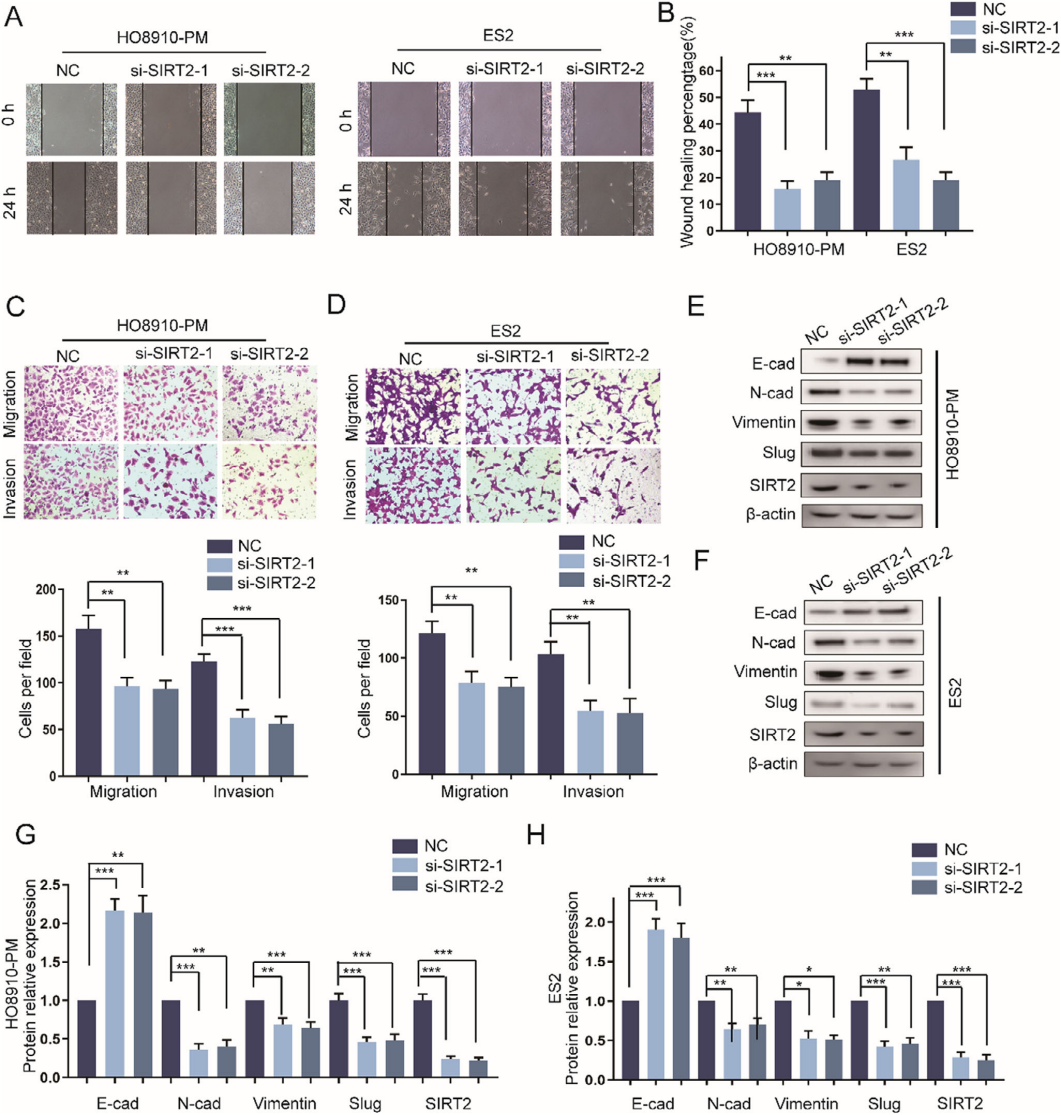
Silencing SIRT2 inhibits the invasion and metastasis of EOC cells. A–D) Cell migration and invasion were examined by wound healing assay and transwell assay in HO8910‐PM and ES2 cells transfected with the SIRT2 siRNA (n = 3 per cell line, Transwell scale bar  =  100 µm, Wound healing scale bar  =  200 µm). E–H) Western blot analysis detecting the EMT makers in HO8910‐PM and ES2 cells with transfected with the SIRT2 siRNA(n = 3). Unpaired Student's *t*‐test was used to compare two groups, one‐way ANOVA was used to compare multiple groups, and Chi‐square test was applied for comparisons of proportions. ^*^ *p* < 0.05, ^**^ *p* < 0.01, ^***^ *p* < 0.001.

The effects of SIRT2 on the metastasis of EOC is still unknown, thus we investigate whether targeting SIRT2 could inhibit the aggressiveness of EOC. Wound healing assay showed that silencing SIRT2 expression significantly attenuated the ability of migration of HO8910‐PM and ES2 cells (**Figure** **6**A,B). Accordingly, transwell assays further validated that knockdown of SIRT2 expression inhibits the migration and invasion of HO8910‐PM cells (Figure 6C), and knockdown of SIRT2 expression suppressed metastasis of ES2 cells (Figure 6D). Furthermore, we determined the effect of the down‐regulation of SIRT2 on the expression of EMT makers. Western blot assay showed that down‐regulation of SIRT2 promotes the expression of E‐cad and reduces the expression of N‐cad, Vimentin, and Slug in HO8910‐PM cells (Figure 6E,G). Similar results were observed in ES2 cells (Figure 6F,H).

### 2.7 Fn14 Alleviates the Metastasis of EOC Cells In Vivo

**Figure 7:**
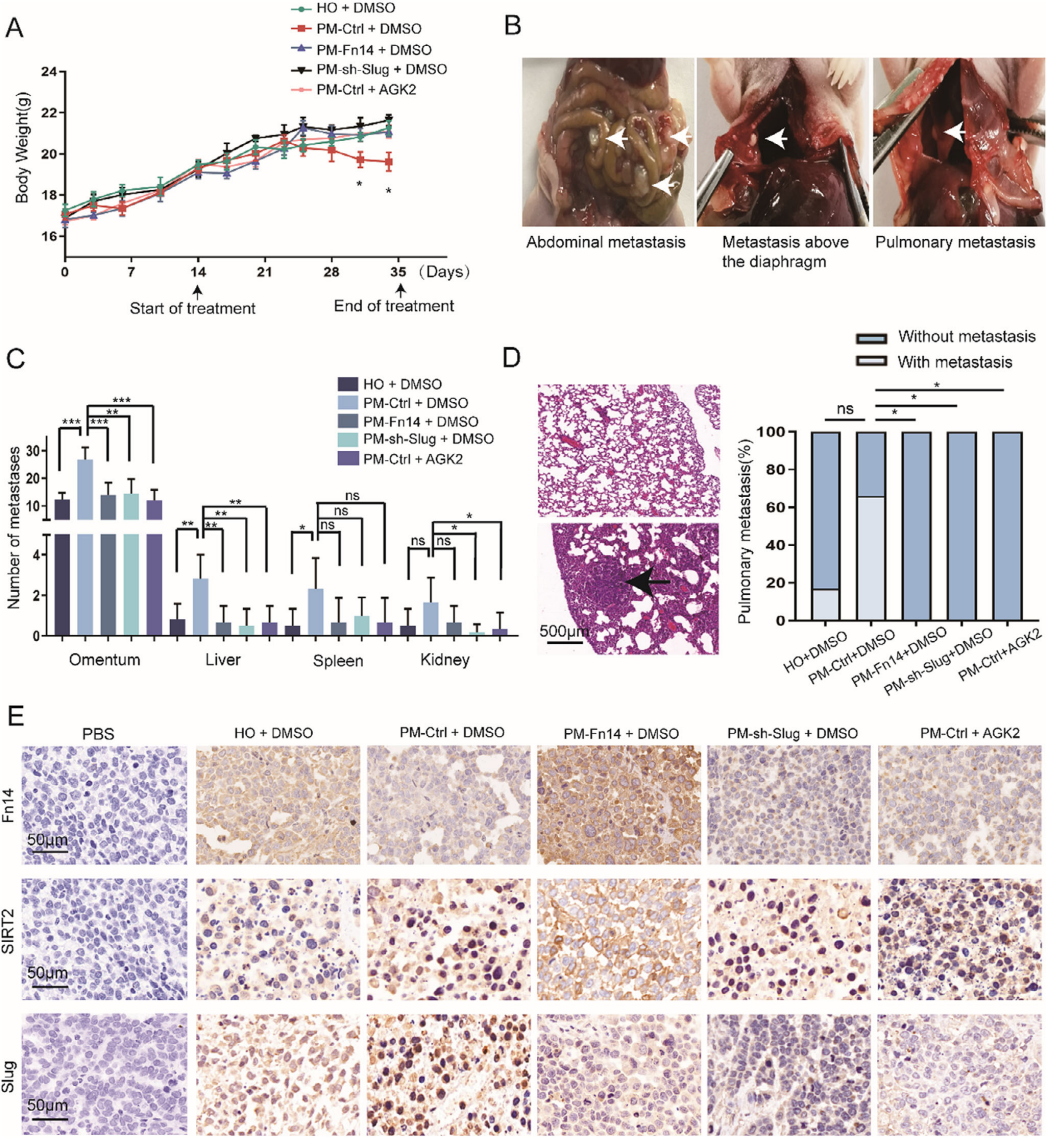
Fn14 alleviates the metastasis of EOC cells in vivo. A) Body weight of mice with HO8910(HO+DMSO), HO8910‐PM/Ctrl (PM‐Ctrl+DMSO), HO8910‐PM/Fn14(PM‐Fn14+DMSO), HO8910‐PM/shSlug (PM‐shslug+DMSO), HO8910‐PM/AGK2(PM‐Ctrl+AGK2) implant are shown. B,C) Metastasis of tumor in five groups are shown. D) Representative hematoxylin–eosin staining and summarized data on lung foci in five groups. E) Tumors of each group were immunohistochemically tested for Fn14, SIRT2, and Slug. One‐way ANOVA was used to compare multiple groups and the Chi‐square test was applied for comparisons of proportions. ^*^ *p* < 0.05, ^**^ *p* < 0.01, ^***^ *p* < 0.001.

To investigate the contribution of Fn14 to metastasis in vivo, nude mice were intraperitoneally injected with different treated EOC cells. We found the mice with HO8910‐PM/Ctrl implant had a significantly lowest body weight compared with HO8910‐PM/Fn14, HO8910‐PM/shSlug, HO8910‐PM/AGK2, and HO8910 implant (**Figure** **7**A). Three weeks following the intraperitoneal injection of these cells into nude mice, metastatic burden was assessed at necropsy (Figure 7B; Figure S5A, Supporting Information). Fn14 overexpression significantly alleviated metastatic burden in mice, including decreasing ascites volume and the number of metastatic nodules within the abdominal cavity (Figure 7C). Similarly, the knockdown of Slug or suppression of SIRT2 activity also showed a decreased metastatic burden in mice. We further observed fewer lung metastasis in nude mice treated with Fn14 overexpression, Slug knockdown, and SIRT2 activity suppression of HO8910‐PM cells, when compared with the control group (Figure 7D). The IHC data further validated that Fn14 overexpression remarkably lessens the SIRT2 protein entry into the nucleus and effectively inhibits the expression of Slug in tumor tissues than that in control tumor tissues (Figure 7E). In addition, tumors from overexpression Fn14 mice showed enhanced expression of E‐cad and reduced expression of N‐cad and Vimentin than tumors from control mice (Figure S5B,C, Supporting Information). Similar results were observed in the mice with Slug knockdown and SIRT2 activity suppression compared to control mice (Figure S5B,C, Supporting Information). Taken together, the above data suggest that Fn14 greatly attenuated the metastatic capacity of ovarian cancer in vivo.

### 2.8 Fn14/SIRT2/Slug Axis Correlates with Prognosis of EOC Patients

**Figure 8:**
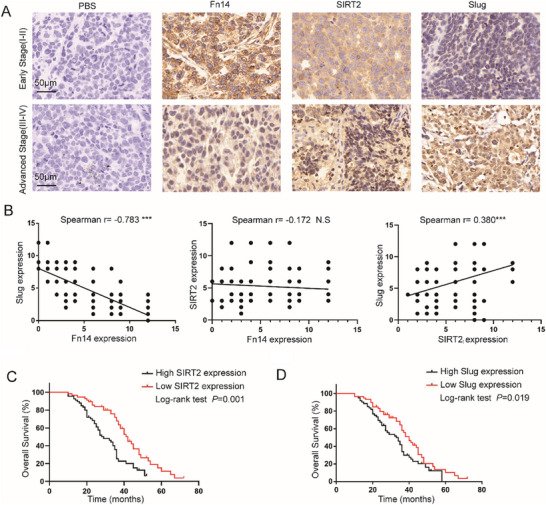
Fn14‐SIRT2‐Slug axis correlates with the prognosis of EOC patients. A) IHC was performed to detect the expression of Fn14, SIRT2, and Slug in EOC samples. B) Spearman's correlation analysis was performed to examine the correlation between the expression levels of Fn14, SIRT2, and Slug in EOC samples. C,D)The correlation between SIRT2 and Slug expression and patient survival was demonstrated in the survival analysis. ^*^ *p* < 0.05, ^**^ *p* < 0.01, ^***^ *p* < 0.001.

To examine the clinical relevance of the Fn14‐SIRT2‐Slug axis in EOC patients, we further analyzed the panel of 122 EOC cases and performed immunohistochemistry to detect the expression of SIRT2 and Slug. Notably, the positive expression of SIRT2 was mainly located in the cytomembrane and cytoplasm rather than in the nucleus of the tumor specimen with high Fn14 expression compared to those with low Fn14 expression, followed by the down‐regulation of Slug expression in the cells (**Figure** **8**A). And Spearman's rank correlation analysis indicated that the expression levels of Fn14 and Slug were inversely correlated and Slug expression was positively correlated with sirt2 expression, whereas SIRT2 expression had no correlation with Fn14 expression (Figure 8B). Moreover, analysis of the TCGA ovarian cancer cohort further validated the correlations between *Fn14*, *SIRT2*, and *Slug* mRNA expression levels (Figure S6A, Supporting Information). In addition, the high expression of SIRT2 and Slug was significantly associated with poor OS (Figure 8C,D). Subsequent analysis of TCGA data revealed that elevated SIRT2 and Slug mRNA levels were strongly associated with worse overall survival, whereas Fn14 mRNA expression showed no statistically significant prognostic relevance (Figure S6B, Supporting Information). The observed discrepancy between the prognostic values of Fn14 mRNA and protein may be related to the fact that mRNA abundance alone does not solely determine protein levels, implicating post‐transcriptional regulation, translational efficiency, or protein stability as critical determinants of Fn14 protein expression. Furthermore, the presence of heterogeneity among patient cohorts, such as varying histological subtypes and treatment regimens, is a potential contributing factor to the observed differences. To validate these findings, we plan to expand our cohort size and perform integrated proteomic profiling in future studies. Together, the data from cancer patients indicated that targeting for Fn14 ‐SIRT2‐Slug axis might be a novel strategy for the treatment of EOC.

## 3 Discussion

Metastasis is the ultimate and most lethal manifestation of cancer.^[^ ^24^ ^]^ The patients are commonly diagnosed at the late stage with implantation metastasis to the pelvis and abdominal cavity, therefore, most of these patients die as a consequence of their metastatic disease and not due to primary tumors.^[^ ^25^ ^]^ Researchers and clinicians carried out extensive studies to explore the molecular mechanism of metastasis, expecting to find more effective therapeutic targets to improve the prognosis of EOC patients. In the present study, our findings showed that EOC patients in the late stage exhibit low Fn14 expression compared to those in the early stage, accompanied with a shorter PFS and OS. Further, our results confirmed that Fn14 inhibits EOC cell migration and invasion both in vitro and in vivo. Mechanistically, Fn14 suppresses the EOC cells metastasis by reducing the Slug‐mediated EMT. Moreover, Fn14 down‐regulated the Slug protein expression by enhancing the acetylation degradation of Slug by interaction with SIRT2. Thus, our study uncovered a novel insight into metastasis in EOC and carried important therapeutic implications.

Emerging evidence has shown that Fn14 contributes to regulating wound repair, inflammation, angiogenesis, migration, and invasion.^[^ ^11^ ^]^ Further, Fn14 exerts tumor suppressive activity by promoting apoptosis in endometrial cancer, colon carcinoma, and hepatocellular carcinoma.^[^ ^26^, ^27^, ^28^ ^]^ In addition, our previous study also revealed that Fn14 reverses chemoresistance by promoting the degradation of TP53‐R248Q in ovarian cancer. However, the effect of Fn14 on metastasis in EOC still remains to be clarified. In our study, patients with low expression of Fn14 were more likely to present at a late stage with longer PFS and OS. Moreover, we further data validated that Fn14 significantly inhibited EOC cell migration and invasion in vivo and in vitro. Therefore, these results strongly indicated Fn14 as a potential therapeutic target in tumor metastasis and a candidate biomarker for prognosis in EOC.

Fn14 has been implicated that modulating invasion and migration of cancer cells mainly rely on activation of downstream signal transduction through TNF receptor–associated factor 2, Rho guanosine triphosphatase, and guanine nucleotide exchange factor.^[^ ^29^ ^]^ Interestingly, our results revealed that Fn14 regulates migration and invasion in the EOC cells by inhibition of EMT. EMT is a process by which epithelial cells lose but acquire mesenchymal characteristics, leading to the acquisition of migratory and invasive traits.^[^ ^30^ ^]^ EOC metastasizes predominantly by peritoneal seeding, a process that is critically driven by the EMT pathway. By activating EMT, tumor cells acquire the ability to detach from the primary tumor, survive in the ascites fluid, and ultimately implant onto the surfaces of the pelvic and abdominal cavities, thereby establishing metastatic lesions. Therefore, Fn14 may serve as a potential therapeutic target for inhibiting ovarian cancer metastasis. EMT can be initiated and promoted by multiple transcription factors, including Snail, Twist, and Zeb family.^[^ ^31^ ^]^ And our further findings suggested that Fn14 remarkably decreases the expression of Slug in EOC, resulting in the inhibition of EMT‐related metastasis. To this end, further studies are needed to elucidate the possible mechanism of how Fn14 modulates Slug expression in EOC.

Moreover, we observed that the down‐regulation of Slug protein mediated by Fn14 is not a result of transcriptional repression. To date, the molecular basis of Slug protein degradation in EOC has remained unclear. Current studies have confirmed that Slug, as a short‐lived protein, is regulated by post‐translational modifications. Taken together, these data suggested that Fn14 regulates Slug expression at post‐translational level. Slug undergoes post‐translational modifications, such as ubiquitination and acetylation, which critically modulate its stability.^[^ ^32^ ^]^ And our data supported that Fn14 selectively increases Slug acetylation levels but does not alter its ubiquitination modificationSIRT2 is a NAD^+^‐dependent deacetylase and Zhou et al. first identified that it deacetylates Slug protein at lysine residue K116 to prevent Slug degradation driving the acquisition of aggressive basal‐like phenotypes and promoting tumor growth in breast cancer.^[^ ^18^ ^]^ Likewise, our findings also supported that SIRT2 deacetylates Slug at K116 in ovarian cancer. SIRT2 dynamically shuttles between the cytoplasm and nucleus, and its subcellular localization directly dictates its deacetylase function. SIRT2 dynamically shuttles between the cytoplasm and nucleus, and its subcellular localization dictates the deacetylation of specific substrates. In our study, we first reported that Fn14 is co‐localized with SIRT2 in EOC cells, we thus speculated that Fn14 disrupts SIRT2‐mediated deacetylation of Slug by interacting with SIRT2. Subsequently, further experiments substantiated this hypothesis that Fn14 interaction with SIRT2 directly reduces SIRT2 shuttling to the nucleus to deacetylase and prevents Slug protein degradation in EOC. Moreover, we explored the interaction domain of SIRT2 binding to Fn14, and we identified that SIRT2 binds to the domain aa 102 to 110 in the C‐terminal intracellular region of Fn14. In addition, we also investigated whether targeting SIRT2 protects EOC patients from metastasis. And, the results have shown that targeting SIRT2 significantly inhibits migration and invasion of cancer cells, indicating targeting SIRT2 may be a promising approach to treat EOC.

**Figure 9:**
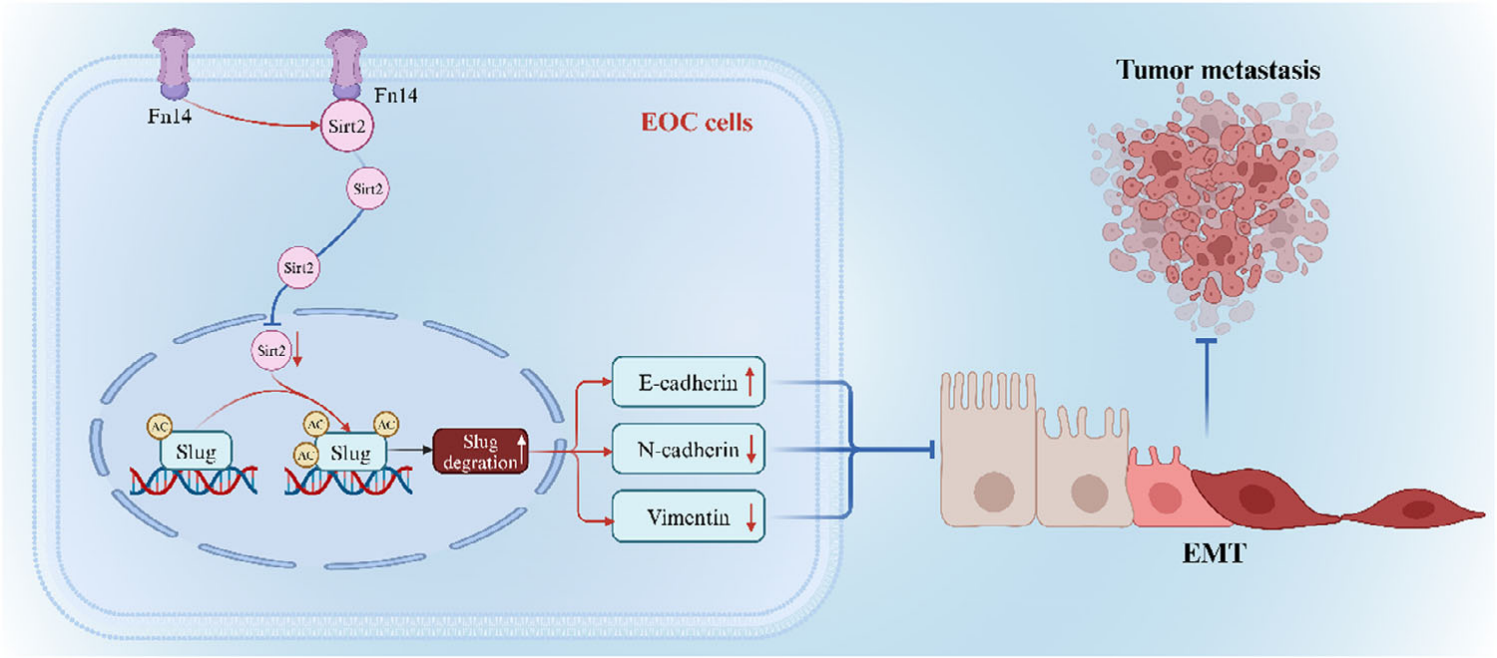
Graphical abstract of Fn14 controls the SIRT2‐mediated deacetylation of Slug to inhibit the metastasis of EOC. Fn14 inhibited EOC metastasis by regulating Slug‐mediated EMT. Furthermore, Fn14 altered the subcellular localization of SIRT2 by interacting with SIRT2, leading to reduced SIRT2 shuttling into the nucleus and subsequently promoting the acetylated degradation of Slug.

In summary, our study for the first time presents a novel role for Fn14 in inhibiting EOC metastasis by regulating Slug‐mediated EMT. The mechanism by which Fn14 regulates Slug expression was identified as Fn14 inhibiting SIRT2 shuttling into the nucleus by interacting with SIRT2, thereby promoting the acetylated degradation of Slug protein (shown in Model in **Figure** **9**). The Fn14/SIRT2/Slug axis provides novel insights into the pathogenesis of EOC, particularly EOC metastasis, and represents a new promising therapeutic target for the treatment of this disease.

## 4 Experimental Section

### Clinical Samples

The study included 122 patients who, between September 2016 and December 2019, underwent ovary debulking surgery at the Affiliated Renji Hospital of Shanghai Jiaotong University, Shanghai, China. All specimens were histologically diagnosed as epithelial ovarian cancer by two experienced pathologists independently. The experimental protocols were approved by the ethical committee of Renji Hospital (RA‐2023‐102), and signed informed consent was obtained from all the patients involved in this study.

### Cell Lines and Chemical Treatments

Human EOC cell lines were purchased from the Cell Bank of the Chinese Academy of Sciences (Shanghai, China). Cells were maintained in PRMI1640 (HyClone, GE Healthcare, UT, USA) media with 10% fetal bovine serum (FBS) (HyClone), 100U/ml penicillin, and 100µg ml^−1^ streptomycin (Sigma Aldrich, St. Louis, MO, USA). For analysis of protein stability, cells were treated with cycloheximide (CHX) (10µM; Selleck Chemicals, Houston, TX, USA) or MG132 (10µM; Selleck Chemicals) followed by pulse‐chase at indicated time points. Selisistat (Selleck Chemicals), AGK2 (Selleck Chemicals), 3‐TYP (Selleck Chemicals), SIRT4‐IN‐1 (MedChemExpress (MCE), NJ, USA), SIRT4‐IN‐1 (MCE), SIRT5 inhibitor 4 (MCE), OSS_128167 (MCE), SIRT7 inhibitor 97491 (MCE) were used to inhibitSIRT family. Leptomycin B was used to inhibit protein nuclear export (10nM, MCE).

### Immunohistochemistry (IHC)

IHC was performed as previously described.^[^ ^13^ ^]^ Slides were reviewed by two pathologists independently, in duplicate. The percentage of positive cells were scored as 0, < 5%; 1, 5–25%; 2, 25–50%; 3, 50–75%; and 4, > 75%. And intensity of staining was scored as 0, negative; 1, weak; 2, moderate; and 3, strong. Scores were multiplied for a final score in the range of 0–12. Scores of 0–4 were defined as low expression, whereas scores of 5–12 were defined as high expression. In addition, antibodies were diluted as follows: anti‐Fn14 (1:500, no.ab109365; Abcam, Cambridge, MA, USA), anti‐Slug (1:100, no. NBP2‐03886; Novus, Littleton, CO, USA), anti‐SIRT2 (1:1000, no.NBP1‐87039; Novus), anti‐E‐cadherin (1:200, no.14472; Cell Signaling Technology (CST), Beverly, MA, USA), anti‐N‐cadherin (1:200, no. 13116; CST) and anti‐Vimentin (1:400, no. 5741; CST).

### Immunoblotting and Immunoprecipitation

Isolation of total proteins from cells was performed using RIPA buffer (10 mM Tris, 150 mM NaCl, 1 mM EDTA, 0.1% SDS) supplemented with a protease inhibitor cocktail. Additionally, cytoplasmic and nuclear proteins were extracted using a nuclear and cytoplasmic extraction kit (Beyotime, Shanghai, China). BCA protein assay kit (Bio‐Rad Laboratories, Hercules, CA, USA) was used to measure the protein concentration of the cell lysate. Immunoblotting was performed according to standard procedures. Protein samples were separated by SDS‐PAGE (8% or 12%), transferred onto a PVDF membrane, and blocked with 5% defatted milk for 30 min. Antibodies were diluted as follows: anti‐Fn14 (1:1000, no.4403; CST), anti‐SIRT2 (1µg ml^−1^, no.s‐8447; Sigma–Aldrich), anti‐Slug (1:1000, no.9662; CST), anti‐E‐cadherin (1:1000, no.3195; CST), anti‐N‐cadherin (1:1000, no.4061; CST), anti‐Vimentin (1:1000, no.5741; CST), anti‐Snail(1:1000, no. 3879; CST), anti‐Twist(1:1000, no. 90445; CST), anti‐GFP(1:1000, no. 2956; CST), anti‐ LaminB1 (1:1000, no. 13435;CST), and β‐actin (1:1000, no. 4970; CST).

For immunoprecipitation experiments, 800 µg of protein extract was incubated with primary antibody overnight. Complexes were then bound to agarose beads for 2 h, washed three times with lysis buffer, eluted by boiling in SDS loading buffer, and performed immunoblotting as indicated above. Antibodies were diluted as follows: anti‐Fn14 (1:50, no.4403; CST), anti‐SIRT2 (25µg ml^−1^, no. 506611; R&D Systems, Minneapolis, MN, USA) and anti‐Acetylated‐Lysine (1:100, no.9441; CST). The recombinant protein of Fn14‐Fc was purchased from MCE and the recombinant protein of His‐SIRT2 was purchased from Fine‐test (Wuhan, China).

### Immunofluorescence Staining

Immunofluorescence staining was performed according to standard procedures. The cells were fixed with paraformaldehyde for 30 min and then incubated for 1 h in a blocking buffer (1% BSA and 0.1% Triton X‐100). Next, the cells were incubated with antibodies against Fn14 (1:50, no. sc‐56250, Santa Cruz), SIRT2 (1:400, no. ab19388; Abcam), and Slug (1:400, no.9585; CST) at 4 °C overnight. The cells were then washed three times with PBS and incubated with fluorescent secondary antibodies for 1 h. The cells were counterstained with DAPI for 15 min at room temperature. A confocal microscope (Nikon, Tokyo, Japan) was used to observe all stained slices.

### Quantitative Real‐Time PCR (RT‐qPCR)

RT‐qPCR was performed as previously described.^[^ ^13^ ^]^ The GAPDH gene was used as an internal control and 2^−ΔΔ^CT was used to compute the expression value of target genes. Primer sequences for RT‐qPCR:

Slug forward: 5′‐CCTCCAAAAAGCCAAACTACTA‐3′

Slug reverse: 5′ ‐GTGTGCTACACAGCAGCC‐3′

Fn14 forward: 5′‐CCAAGCTCCTCCAACCACAA‐3′

Fn14 reverse: 5′‐TGGGGCCTAGTGTCAAGTCT‐3′

GAPDH forward: 5′‐ATCACCATCTTC CAGGAGCGA‐3′

GAPDH reverse: 5′‐CCTTCTCCATGGTGGTGAAGAC‐3′

### RNA‐Sequencing and Gene Expression Analysis

Total RNA of cells was extracted using RNeasy Plus Kit (Qiagen, GmbH, Hilden, Germany) according to the manufacturer's protocol. Total RNAs with RNA Integrity Number (RIN) of >8 were subjected to full‐length transcriptome sequencing. Illumina Hiseq X Ten platform was used to generate 125 bp paired‐end reads (Amplicongene, Shanghai, China). The raw data were filtered for quality control of the reads with the fast QC package and then aligned to the Ensembl GRCh37/hg19 human reference using TopHat v2.0.9. Cufflinks (v2.1.1) were used to count the reads mapped to each gene. Differential expression analysis and FPKM (fragments per kilo‐base million) calculation of genes in each sample were performed by edgeR. Transcripts with adjusted p‐values of <0.05 were assigned as differentially expressed genes.

### Transient Transfection

Fn14 siRNA and Slug siRNA were purchased from Integrated Biotech Solutions (Shanghai, China). SIRT2 siRNA was purchased from Santa Cruz (sc‐40988). Cells were transiently transfected using Lipofectamine 2000(Invitrogen, Carlsbad, CA, USA). Fn14, Sense‐1: 5′‐CAUCCAUUCUAGAGCCA‐GUCUTT‐3′, Sense‐2 5′‐GAGGGA‐GAAUUUAUUAAUAAATT‐3′, Slug, Sense‐1: 5′‐CCGUAUCUCUAUGAGAG‐UUACUCCA‐3′, Sense‐2: 5′ ‐GAUGCAUAUUCGGA‐CCCACACAUUA‐3′.

### Lentiviral Infections

The overexpression Fn14 lentivirus and Slug shRNA lentivirus were purchased from the Gikai gene (Shanghai, China). The lentivirus was introduced into HO8910‐PM cells by adding to the cell growth medium. Then, stably overexpressing Fn14 or silencing Slug cells were selected using a medium containing 1 µg ml^−1^ puromycin (Sigma–Aldrich).

### Cell Adhesion Assays

Exponentially growing cells were seeded in 96‐well plates coated with vitronectin (Sigma, Germany). After being incubated for 2, 4, 8, and 12 h at 37 °C, the non‐adherent cells were removed using PBS. Adherent cells were fixed with CCK‐8 reagent (10µl well^−1^, Dojindo, Tokyo, Japan) and incubated for 3 h at 37 °C. The adhesion of cells was determined using the cell counting kit 8 (CCK8) according to the manufacturer's protocol (Dojindo Molecular Technologies, Kumamoto, Japan).

### Wound Healing, Migration, and Invasion Assays

Wound healing, migration, and invasion assays were performed as described previously.^[^ ^33^ ^]^

### Animal Experiment

Animal protocols were in accordance with the Shanghai Medical Experimental Animal Care Guidelines. The research was approved by the Institutional Animal Care and Use Committee of Shanghai Jiao Tong University School of Medicine (2023‐180). Five‐weeks‐old BALB/c nude mice were randomly allocated into five groups (n = 6 per group). Then the mice were intraperitoneally injected with different treated EOC cells (2.5×10^6^ cells). AGK2 (10 mg kg^−1^, Selleck Chemicals) was administered intraperitoneally twice a week up for to 4 weeks, and the other groups received an equal volume of DMSO. After six weeks, mice were euthanized and tumors were harvested for further analyses.

### Bioinformatic Analysis

Gene expression and survival analyses of *Fn14, SIRT2, and Slug* in ovarian cancer tissues (n = 381) were performed using RNA‐sequencing (RNA‐seq) and clinical data from The Cancer Genome Atlas (TCGA) database (https://portal.gdc.cancer.gov/). Survival analysis employed Kaplan‐Meier curves with log‐rank tests, stratifying patients into high/low expression groups based on median *Fn14/SIRT2/Slug* expression or optimized cutoff values.

### Statistical Analysis

The analyses were performed using Prism 8.0 software (GraphPad, Inc., San Diego, CA, USA). The results were presented as mean ± SD for three independent experiments. For continuous variables, a two‐tailed Student's *t*‐test was performed for comparison between two groups, and a one‐way analysis of variance (ANOVA) was performed for multiple group comparisons. For categorical variables, Fisher's exact test or Chi‐square test was used for the detection of statistical differences. A nonparametric test was used when data deviated from normality or heteroscedasticity. Values of *p* < 0.05 were considered as statistically significant.

## Conflict of Interest

The authors declare no conflict of interest.

## Author Contributions

A.‐Y.W., S.L., and C.F. Co‐first authors and these authors contributed equally to this work. L.H.Q. and L.H. designed and supervised this study. A.Y.W., C.Y.F., and S.Z.L. conducted experiments. A.Y.W., J.H.H., and W.J.W performed data analysis. Z.J.H. and R.L.W. collected the EOC samples and clinical information. A.Y.W., L.H.Q., and L.H. wrote the manuscript with input from all the authors.

## Supporting information



Supporting Information

## Data Availability

Data sharing is not applicable to this article as no new data were created or analyzed in this study.
