# Supplementary material for: Fn14 Controls the SIRT2‐Mediated Deacetylation of Slug to Inhibit the Metastasis of Epithelial Ovarian Cancer
Source: Adv Sci (Weinh). 2025 May 8;12(27):2501552. doi: 10.1002/advs.202501552 (PMC12279162; doi:10.1002/advs.202501552)
Supplement: Supplementary file 1 — Supporting Information [file ADVS-12-2501552-s001.docx]

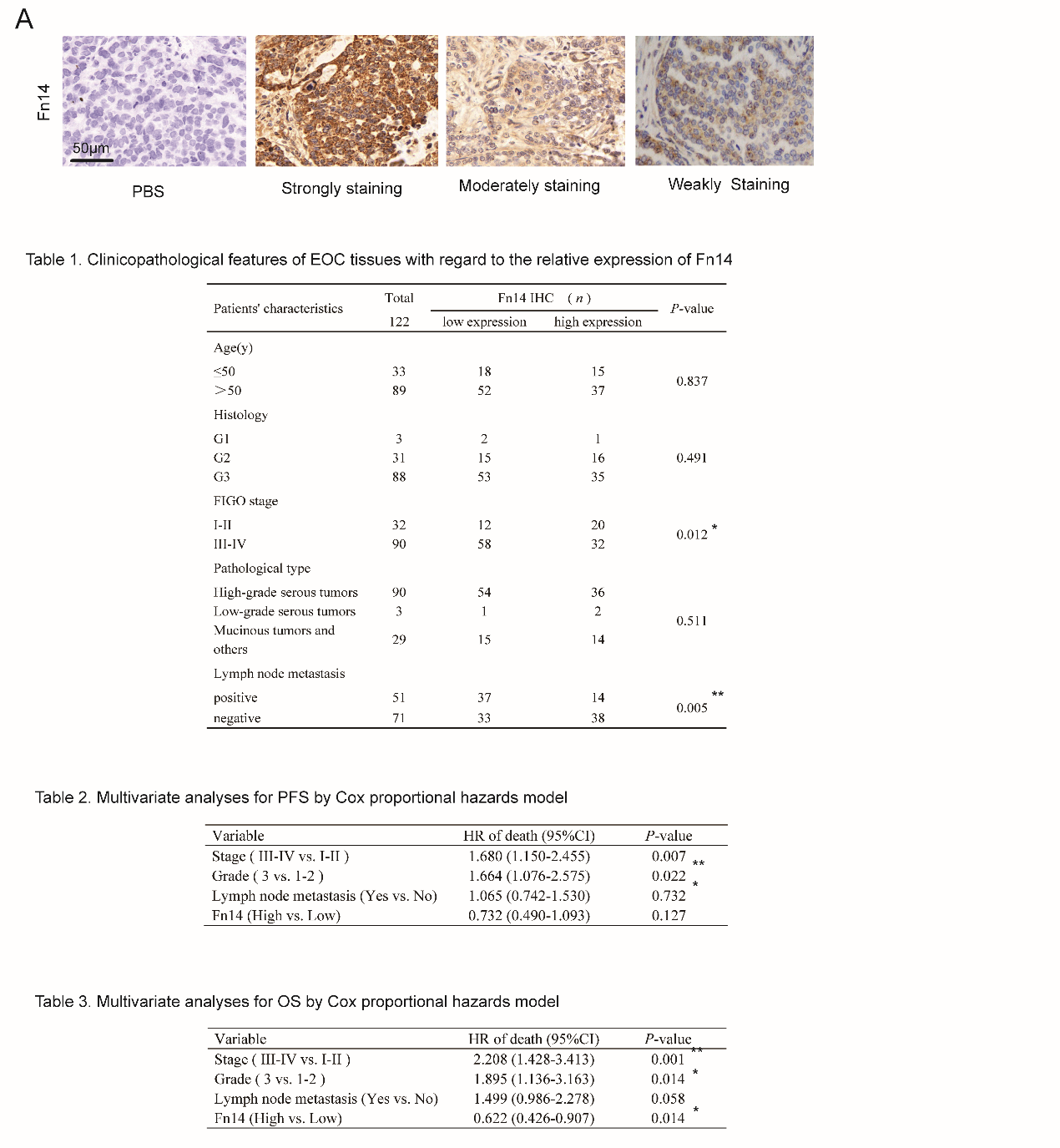


**Figure S1. Loss of Fn14 correlates with metastasis and poor prognosis of EOC**

(A) Fn14 expression levels in 122 EOC samples, as determined by IHC. ^*^*p* < 0.05, ^**^*p* < 0.01, ^***^*p* < 0.001.

**
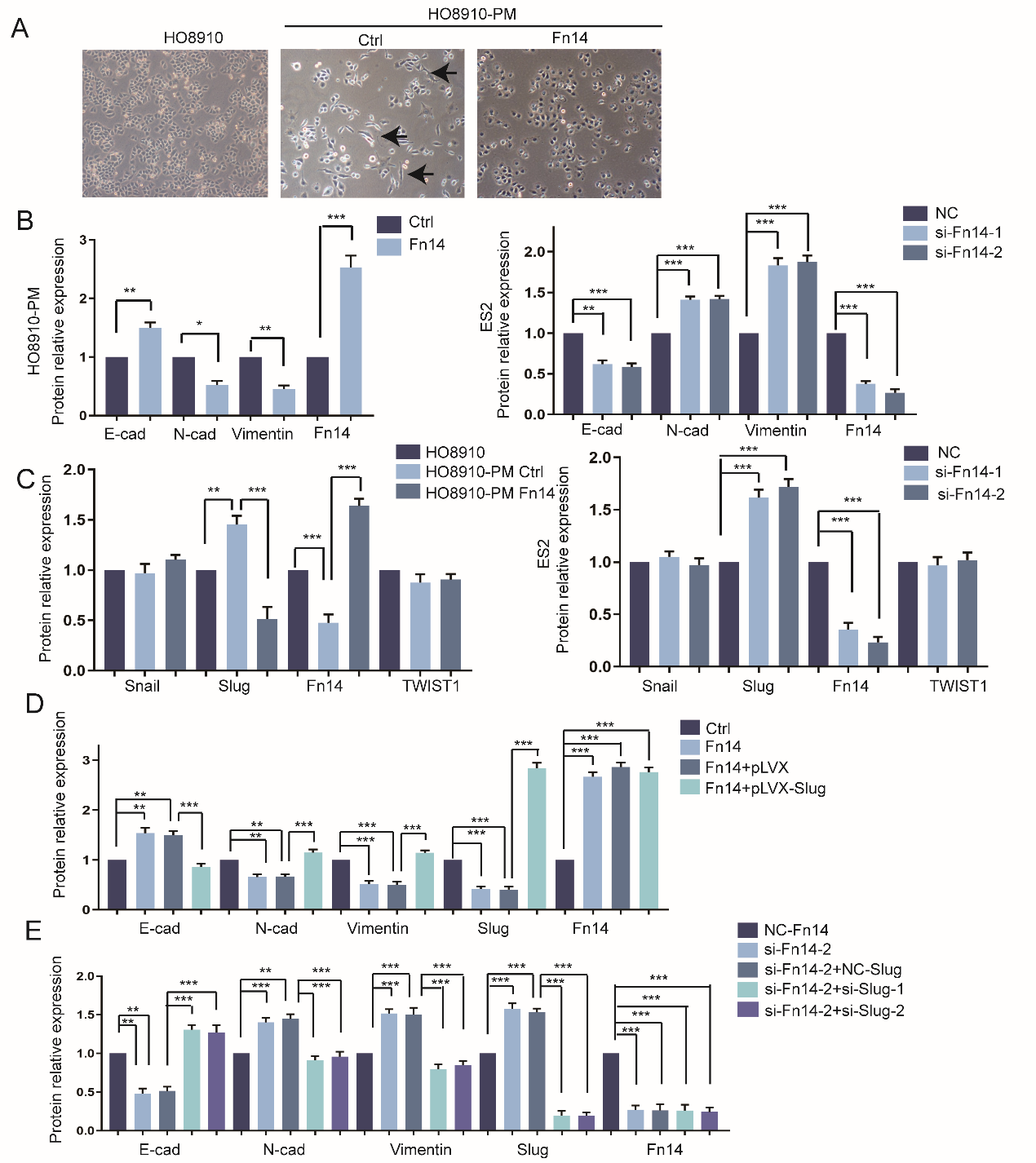
**

**Figure S2. Fn14 attenuates metastasis of EOC cells by regulating EMT**

(A) Overexpression of Fn14 changes the cell morphology of HO8910-PM cells, magnification of 200×. (B)-(E) Statistical data of western blot. ^*^*p* < 0.05, ^**^*p* < 0.01, ^***^*p* < 0.001.

**
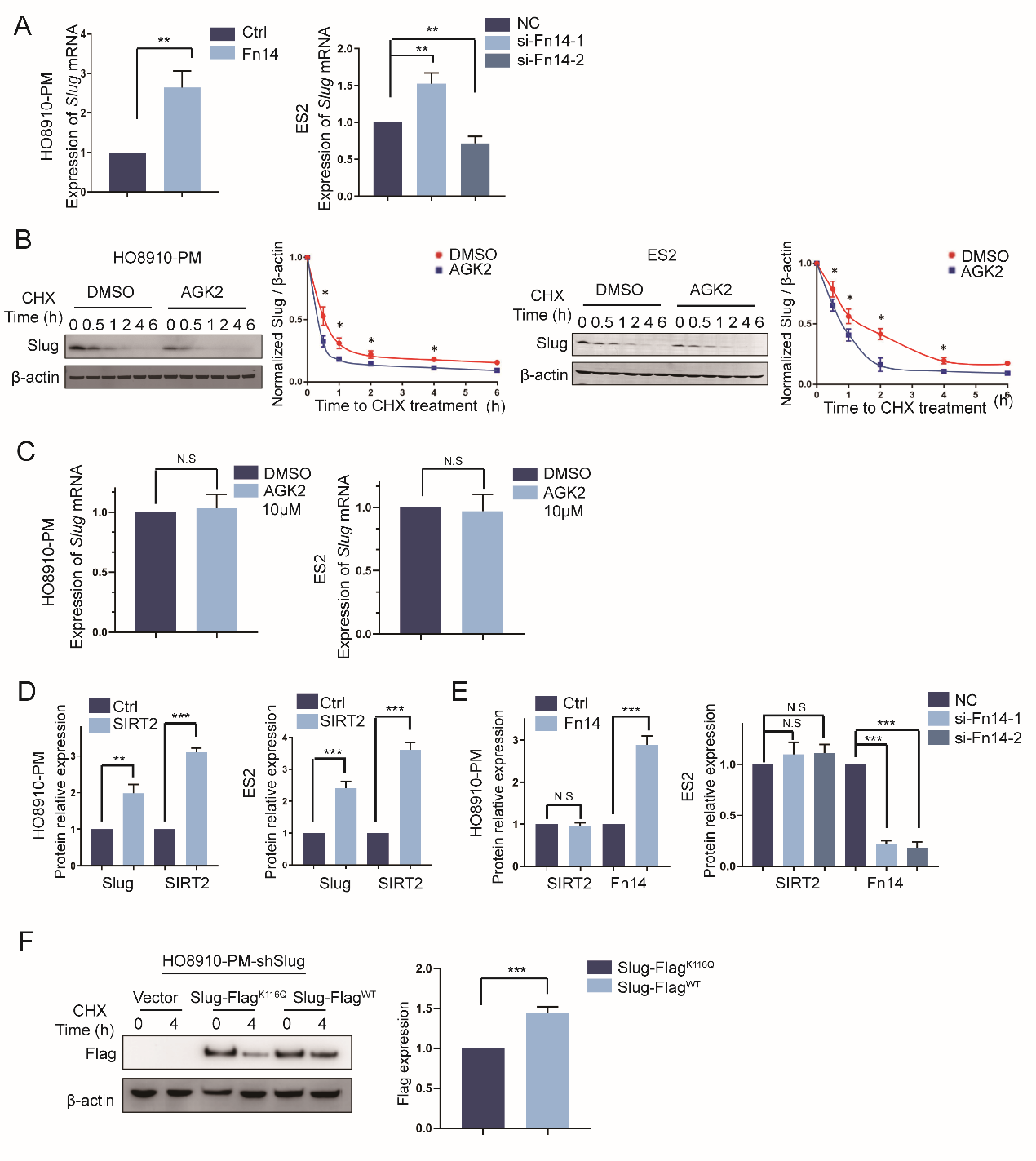
Figure S3. Fn14 promotes the degradation of Slug protein**

(A) RT-qPCR detecting the mRNA expression of *Slug* in HO8910-PM and ES2 cells with specific treatment (n=3). (B) Upon AGK2 treatment, western blot analysis detecting the expression of Slug in EOC cells (n=3). (C) The working concentration of AGK2 for selection and RT-qPCR detecting the mRNA expression of *Slug* in EOC cells with AGK2 treatment (n=3). (D) and (E) Statistical data of western blot. (F) The effect of mutating Slug's K116 on the stability of Slug was detected by western blot in the HO8910-PM knockdown Slug using shSlug lentivirus (n=3) ^*^*p* < 0.05, ^**^*p* < 0.01, ^***^*p* < 0.001.


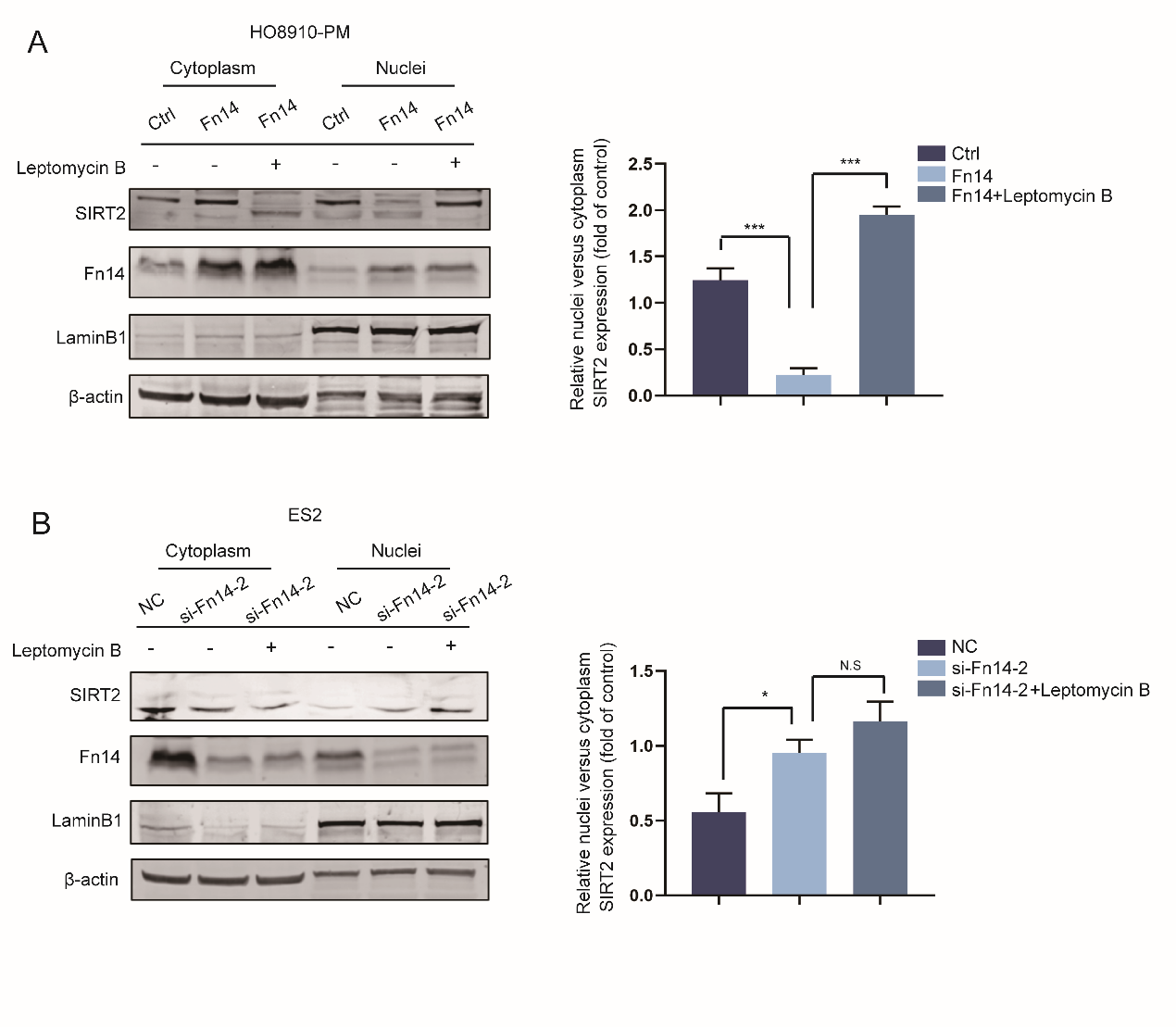


**Figure S4. Fn14 affects the subcellular localization of SIRT2**

(A) and (B) Upon Leptomycin B treatment, detecting the distribution of SIRT2 in cytoplasm or nucleus in EOC cells with up- or down-regulated expression of Fn14 by western blot analysis (n=3) in HO1089-PM and ES2 cells. ^*^*p* < 0.05, ^**^*p* < 0.01, ^***^*p* < 0.001.


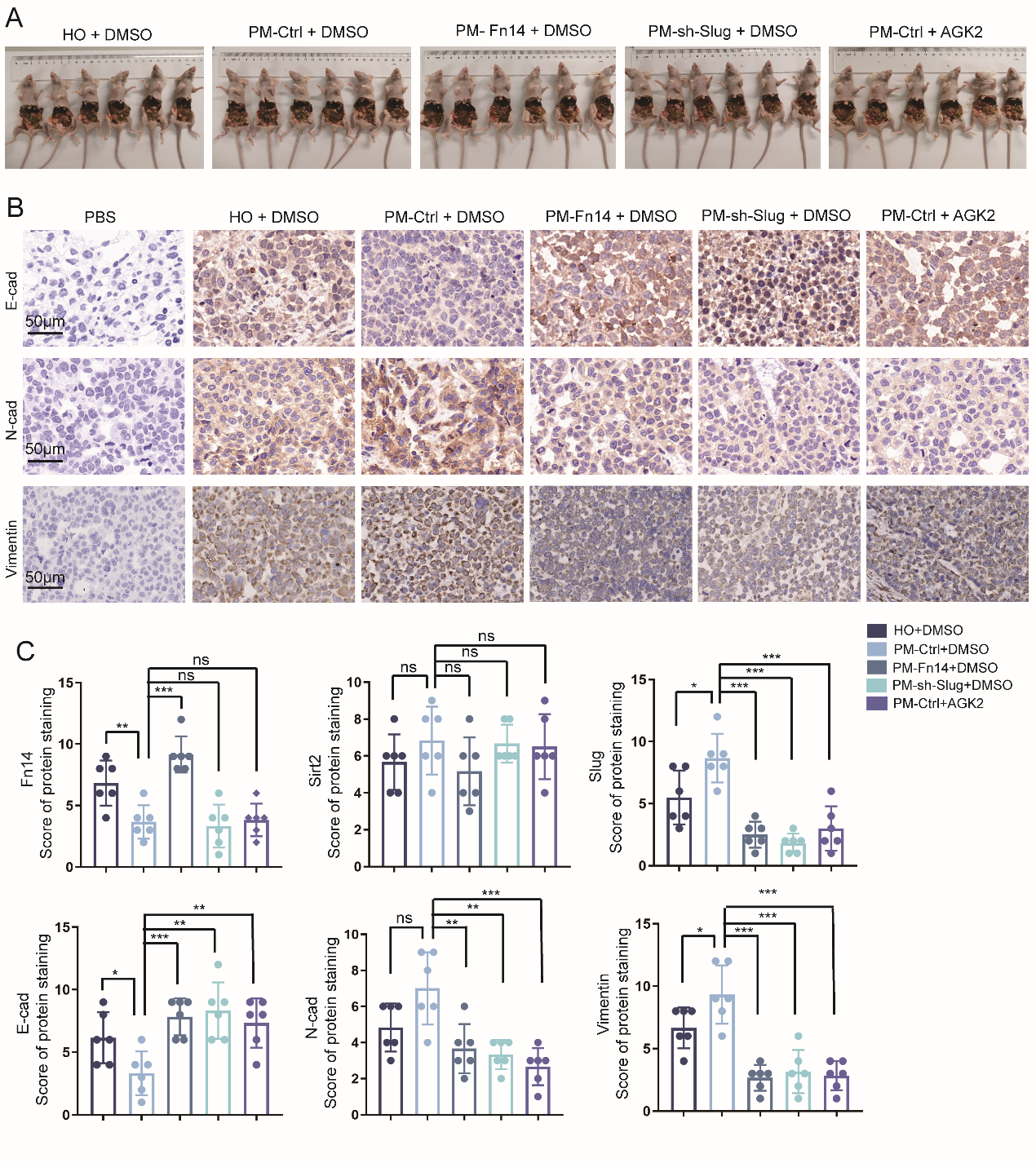


**Figure S5. Fn14 alleviates the metastasis of EOC cells in vivo.**

(A) Representative images of mice with different treatment. (B) Tumors of each group were immunohistochemically tested for Fn14, Sirt2 and Slug. (C) Statistical data of IHC. ^*^*p* < 0.05, ^**^*p* < 0.01, ^***^*p* < 0.001.


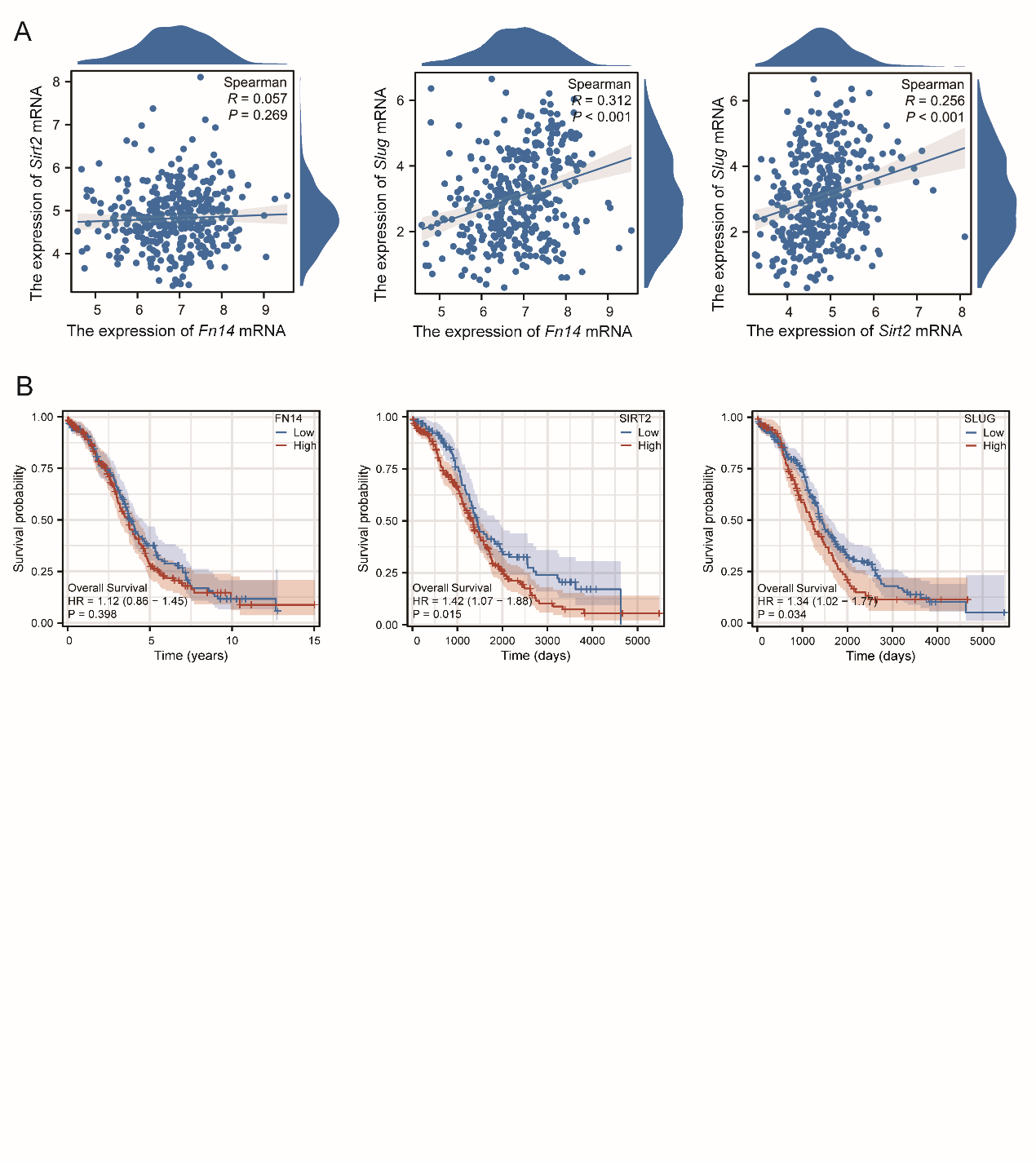


**Figure S6. Bioinformatic analysis of the correlation between Fn14, SIRT2 and Slug mRNA expression and prognosis in ovarian cancer.**

(A) Correlation between Fn14, Sirt2 and Slug mRNA expression (Data from TCGA, n=381). (B) Association analysis between Fn14, Slug and SIRT2 mRNA expression levels and prognosis in ovarian cancer patients. ^*^*p* < 0.05, ^**^*p* < 0.01, ^***^*p* < 0.001.
